# Evolution and spread of Venezuelan equine encephalitis complex alphavirus in the Americas

**DOI:** 10.1371/journal.pntd.0005693

**Published:** 2017-08-03

**Authors:** Naomi L. Forrester, Joel O. Wertheim, Vivian G. Dugan, Albert J. Auguste, David Lin, A. Paige Adams, Rubing Chen, Rodion Gorchakov, Grace Leal, Jose G. Estrada-Franco, Jyotsna Pandya, Rebecca A. Halpin, Kumar Hari, Ravi Jain, Timothy B. Stockwell, Suman R. Das, David E. Wentworth, Martin D. Smith, Sergei L. Kosakovsky Pond, Scott C. Weaver

**Affiliations:** 1 Institute for Human Infections and Immunity, Department of Pathology and Department of Microbiology and Immunology, University of Texas Medical Branch, Galveston, Texas, United States of America; 2 Department of Medicine, University of California, San Diego, La Jolla, California, United States of America; 3 Virology Group J. Craig Venter Institute, Rockville, Maryland, United States of America; 4 cBio, Fremont, California, United States of America; 5 Informatics Group, J. Craig Venter Institute, Rockville, Maryland, United States of America; 6 Bioinformatics and Systems Biology Graduate Program, University of California, San Diego, La Jolla, California, United States of America; Yale University, UNITED STATES

## Abstract

Venezuelan equine encephalitis (VEE) complex alphaviruses are important re-emerging arboviruses that cause life-threatening disease in equids during epizootics as well as spillover human infections. We conducted a comprehensive analysis of VEE complex alphaviruses by sequencing the genomes of 94 strains and performing phylogenetic analyses of 130 isolates using complete open reading frames for the nonstructural and structural polyproteins. Our analyses confirmed purifying selection as a major mechanism influencing the evolution of these viruses as well as a confounding factor in molecular clock dating of ancestors. Times to most recent common ancestors (tMRCAs) could be robustly estimated only for the more recently diverged subtypes; the tMRCA of the ID/IAB/IC/II and IE clades of VEE virus (VEEV) were estimated at ca. 149–973 years ago. Evolution of the IE subtype has been characterized by a significant evolutionary shift from the rest of the VEEV complex, with an increase in structural protein substitutions that are unique to this group, possibly reflecting adaptation to its unique enzootic mosquito vector *Culex* (*Melanoconion*) *taeniopus*. Our inferred tree topologies suggest that VEEV is maintained primarily *in situ*, with only occasional spread to neighboring countries, probably reflecting the limited mobility of rodent hosts and mosquito vectors.

## Author summary

The Venezuelan equine encephalitis (VEE) complex comprises a broadly distributed group of alphaviruses in the Americas that have the potential to emerge and cause severe disease. Historically, VEE complex viruses have caused recurring outbreaks of human and equine encephalitis in Central and South America as well as Mexico, with at least one outbreak resulting in movement of the virus to the southern United States. We present the most comprehensive phylogenetic analysis of complete genomic sequences of the most prominent member of the VEE complex, VEE virus (VEEV). We were able to identify the major forces influencing VEEV evolution, and using the inferred phylogenies we determined that VEEV evolves in geographically segregated lineages with enzootic transmission between rodents and mosquitoes apparently limiting its spread.

## Introduction

The Venezuelan equine encephalitis (VEE) antigenic complex comprises a group of alphaviruses that share similar genetic characteristics and can be defined by broad cross-reactivity antigenically [1] which defines them as a group within the *Alphaviridae*. The VEE complex alphaviruses are classified into six subtypes, designated I to VI, and consist of 9 species [2], of which subtype I contains the veterinary and medically important VEE virus (VEEV) (Table 1). Historically, the VEE complex subtypes I-VI were defined by serological analysis, and this nomenclature has persisted. However, the advent of sequencing resulted in several viruses now being reclassified as distinct species. For example, subtype IF is genetically distinct from the remainder of the subtype I viruses and is now classified as the species *Mosso das Pedras virus*. Most VEE complex viruses have enzootic cycles where they circulate between wild animals, generally rodents and mosquitoes, particularly *Culex* (*Melanoconion*) spp. mosquito vectors. With the exception of VEEV subtype II, VEE complex viruses are geographically distributed throughout Central and South America. VEEV subtype II is found only in Florida and is usually transmitted by *Culex cedeci* mosquitoes. The recent appearance for the first time of *Culex* (*Melanoconion*) species in southern Florida [3] underscores the continuing threat of emergence, possibly enhanced by climate change, which also increases the potential for other VEEV subtypes to spread northwards and establish enzootic transmission cycles. Although many VEE complex viruses have not been implicated in human disease, those that are associated with human disease (VEEV) can cause acute, often severe febrile illness that may progress to encephalitis, causing severe human morbidity and mortality [4]. Patients who survive encephalitis are often left with permanent neurologic sequelae, and the cost for treatment and long-term care related to a single case can be several million dollars [5]. In addition to VEEV(subtype I), which cases the majority of the encephalitis cases within the VEE subtype, subtype II Everglades virus (EVEV), which is found only in Florida, can cause neurologic disease in humans [6] and equids [7]. Subtype IIIA, Mucambo virus, also causes febrile disease in humans [8, 9].

**Table 1:** The subtypes of VEE complex alphaviruses and their transmission cycles.

Virus	Subtype	Abbreviation	First Isolation	Geographic Range	Vertebrate Host Range	Mosquito vector	Human Disease	Endemic/Epidemic
Venezuelan equine encephalitis	IAB	VEEV	Venezuela 1938	Trinidad, Peru, Colombia, Guatemala-Mexico-Texas	Horses/humans	*Aedes* and *Psorophora spp*	Yes	Epidemic
	IC		Colombia, 1962	Colombia, Venezuela, Peru	Horses/humans	*Aedes* and *Psorophora spp*		Epidemic
	ID		Colombia, 1961	South and Central America	Rodents	*Culex (Melanoconion) spp*.		Endemic
	IE		Panama, 1961	Central America	Rodents, horses, humans	*Culex (Melanoconion) taeniopus* and *Psorophora* and *Aedes spp*.		Epidemic/Endemic
Everglades	II	EVEV	Florida, USA, 1963	Florida	Birds	*Culex (Melanoconion spp*.*)*	No	Endemic
Mucambo	IIIA	MUCV	Trinidad, 1954	South America	Unknown	Culex portesi	yes	Endemic
Tonate	IIIB	TONV	French Guiana, 1973	South and Central America	Unknown	Unknown	No	Unknown
71D1252	IIIC			South America	Unknown	Unknown	No	Unknown
Pixuna	IV	PIXV	Brazil, 1964	South America	Unknown	Unknown	No	Unknown
Cabassou	V	CABV	French Guiana, 1968	French Guiana	Unknown	*Culex portesi*	No	Unknown
Rio Negro (AG80_663)	VI	RNV	Argentina, 1980	Argentina	Unknown	Unknown	No	Unknown
Mosso das Pedras (78V3531)	IF	MDPV	Brazil, 1978	Brazil	Unknown	*Culex* (*Melanoconion*) sp.	No	Unknown

VEEV is associated with human disease, and is further subdivided into subtypes IAB, IC, ID, IE. Subtypes ID and IE comprise enzootic/endemic strains that circulate continuously in forests and swamps of northern South America, Central America and Mexico and cause a large burden of endemic disease from direct spillover [10]. The remaining VEEV subtypes, IAB and IC, comprise epizootic/epidemic strains that are associated with periodic equine-amplified outbreaks that result in severe disease in equids and extensive spillover to humans [11]. These outbreaks can spread from South America as far north as the southern United States [12, 13], resulting in up to hundreds-of-thousands of cases over a period of months to a few years. Prior to the 1980s, VEE epizootics involving high case-fatality rates were frequently recorded. Because horses have been an important component of the local agricultural economies within many Latin American regions, VEE has often had a sizeable economic impact as well as a direct effect on public and veterinary health [14]. Recent outbreaks during the 1990s in Venezuela, Colombia and Mexico have demonstrated the potential for VEEV to re-emerge periodically from enzootic progenitors [15–18]. The emergence of VEEV into an epidemic/epizootic form has been associated with specific mutations that arise in the VEEV envelope glycoprotein 2 (E2) gene of enzootic subtype ID or IE strains. These mutations result in the addition of positively charged amino acid changes on the surface of the virion spikes [19] that give rise to increased virulence and viremia in equids [20, 21] and sometimes enhanced infection of epidemic vector mosquitoes such as *Aedes* (*Ochlerotatus*) *taeniorhynchus* [22]. The higher viremia levels in equids can lead to infection of mosquitoes that are not normally involved in enzootic circulation [23], which can then result in spillover infections of humans and other domestic animals.

The phylogenetic characteristics of the VEE complex have been studied for several decades, recently focusing on structural protein gene sequences [24–27]. These studies support the hypothesis that the IAB and IC subtypes arise from mutations in enzootic ID strains that result in the acquisition of epizootic/epidemic characteristics [19, 20, 28]. Although some studies have addressed the evolution and continued circulation of VEEV ID and IE strains, the use of only partial genomic sequences has limited their resolution and accuracy.

To date, insufficient complete genomic sequences have been available to permit a detailed, global analysis of all VEE complex species/strains and obtain high-resolution phylogenetic results. Our goal was to determine more accurately the temporal origin of the VEE complex and patterns of historic spread. By increasing the number of sequenced VEEV strains from the ID and IE subtypes, we sought a more robust analysis of the evolution of these subtypes. To this end, a set of 130 complete genome sequences was prepared, of which 94 were determined in this study, and a comprehensive phylogenetic study was performed to determine the origin and evolutionary patterns of these important viruses.

## Materials and methods

### Virus isolates

Viruses were obtained from the World Reference Center for Emerging Viruses and Arboviruses and other collections at the University of Texas Medical Branch, and included 94 isolates that had not been previously sequenced or had only partial genome sequences available. These strains were geographically and temporally distributed across North and Central America, and over the past 80 years. The metadata for the 94 virus strains sequenced in this study and their accession numbers are found in S1 Table.

### Virus propagation and cDNA generation

Vero (African green monkey kidney) cells (CCL-81, ATCC) were grown in 150 cm^2^ flasks to ~80% confluency and inoculated with VEE complex virus strains. S1 Table shows the strains included in this study and their associated metadata. Infected cells were maintained at 37°C until the development of cytopathic effects. Then, cell culture supernatants were clarified at 1,125 *x g* for 10 min and mixed with a 1/3 volume of 4X precipitation buffer (28% PEG 8000, 9.2% NaCl). After overnight incubation at 4°C, virus was pelleted at 2,880 *x g* for 30 min at 4°C and resuspended in 250 μl of TEN buffer (10 mM Tris-HCl, pH 7.5, 1 mM EDTA, 0.1 M NaCl), which was then added to 750 μl of TRIzol LS Reagent (Invitrogen, Grand Island, NY). RNA was extracted following the manufacturer’s protocol, then resuspended in 50 μl of H_2_O and stored at -80°C.

The SuperScript III First-Strand Synthesis System (Invitrogen, Grand Island, NY) was used to produce cDNA following the manufacturer’s recommendations. Three 20 μl-reactions were performed, each with 6 μl of extracted RNA and 1 μl of one of the following cDNA primers, 50 ng/μl of random hexamers, 50 μM oligo (dT)_20_, and 10 μM of reverse primers designed to anneal approximately 500 nt downstream of the 5’ end of the viral genome. Samples were treated with RNase H, and the resulting cDNA generated from random hexamers and oligo (dT)_20_ primers were then mixed together. The cDNA samples were stored at -80°C until further processing, and efficient reverse transcription was confirmed by PCR using 0.5 μl of each sample and primer pairs designed to anneal near the 5’ and 3’ portions of the genome.

### Sequencing of VEEV genomes

Sequences were assembled using sequence-independent single primer amplification (SISPA) to barcode random primed cDNAs [29, 30] from individual cDNA samples. SISPA products were normalized and pooled into a single reaction that was purified using a PCR purification kit (Qiagen, Valencia, CA). Samples were subsequently gel purified to select for products ranging from 300-500bp in size for sequencing with the Illumina Genome Analyzer II or 500-800bp in size for Roche 454 Titanium (GS-FLX) sequencing [31], or were sequenced on the Illumina HiSeq using the following protocol; cDNA (0.05–1.7 μg) was fragmented by incubation at 94°C for eight (8) minutes in 19.5 ul of fragmentation buffer (Illumina 15016648). Samples were tracked using the “index tags” incorporated into the adapters as defined by the manufacturer. Cluster formation of the library DNA templates was performed using the TruSeq PE Cluster Kit v3 (Illumina) and the Illumina cBot workstation using conditions recommended by the manufacturer. Paired end 50 base sequencing by synthesis was performed using TruSeq SBS kit v3 (Illumina) on an Illumina HiSeq 1500 using protocols defined by the manufacturer.

### Assembly of VEEV genomes

Next generation sequencing (NGS) reads from Roche 454 GS-FLX were sorted based on SISPA barcode matches, trimmed, and searched by TBLASTX against a custom reference nucleotide database of full-length VEE complex genomes downloaded from GenBank. Any chimeric VEEV sequences or non-VEEV sequences amplified during the random hexamer-primed amplification were removed. For each sample, the filtered GS-FLX reads were then *de novo* assembled using CLC Bio’s *clc_novo_assemble* program. The consensus sequence of the *de novo* assembly was used to identify the best full-length VEEV genome downloaded from GenBank to use as a mapping reference sequence. Both GS-FLX and Illumina reads were then mapped to the selected reference VEEV genome using CLC Bio’s *clc_ref_assemble_long* program. At loci where both GS-FLX and Illumina sequence data agreed on a variant compared to the reference sequence, the latter was updated to reflect the difference. A final mapping of all sequences to the updated reference sequences was then performed, resulting in the final assembled genome. Upon review of NGS assemblies, there were circumstances that required the use of RT-PCR followed by Sanger capillary sequencing to fill gaps in genomic regions with low coverage. These cases included finishing/closure tasks to increase the sequencing coverage of genome regions inadequately covered by NGS.

Sequences identified with an asterisk in S1 Table were part of a different sequencing project and were therefore analyzed using a different platform. These sequences were first subjected to a blast analysis to determine the number of viral reads and the percentage of contaminating reads from hosts, or other sources. Then the contamination reads from the Vero cells were filtered out using the African Green Monkey (*Chlorcebus sabeus*) genome as a template. The remaining Fastq files were additionally processed using trimmomatic to remove low quality sequence. Finally, assembly was performed using iMetAMOS [32] using the standard parameters.

### Data sets

Nucleotide sequences were manually aligned with similar GenBank sequences using MUSCLE implemented in SeaView [33]. Untranslated regions (UTRs) of alphavirus genomes show limited conservation and are often difficult to align reliably. Therefore, the sequences were edited and trimmed to remove untranslated regions, resulting in concatenated ORFs for 130 (126 VEEV and 4 EEEV) sequences. Sequences were then re-aligned as protein sequences before being reverse translated to nucleotides to maintain codon alignments. Within the VEEV genome, there are two regions that have proved historically difficult to align, the 3’ end of the nsP3 and the 5’ end of the capsid gene [34]. Thus these were removed from the alignment and the resulting alignment was used for subsequent analyses and this alignment is available upon request. Eastern equine encephalitis virus, the sister virus to VEEV in the alphavirus genus [35], was used as an outgroup in all analyses except molecular dating and selection. Sequences were analyzed for percent identity at the nucleotide and amino acid level using BioEdit [36].

### Phylogenetic trees

A maximum likelihood (ML) phylogeny was inferred using PAUP* 4.0 [37] and the General Time Reversible (GTR +Γ_4_+I) model, selected by Modeltest [38]. To assess the robustness of tree topologies, bootstrapping was performed using 1,000 replicate neighbor-joining trees. Bayesian phylogenetic inference was performed using the GTR +Γ_4_+I model in MrBayes v3.1 [39, 40]. Analyses were run for one million iterations until they reached convergence.

### Estimated evolutionary rates and dates of divergence

Substitution rates and times to most recent common ancestor (tMRCAs) were estimated using Bayesian evolutionary analysis by sampling trees (BEAST) v1.7.1 [41, 42]. BEAST employs a Bayesian Markov chain Monte Carlo (MCMC) approach to infer demographic histories, evolutionary rates and dates of divergence from serially (dated) sampled sequence data. Statistical uncertainty in the data is reflected in the 95% highest posterior density (HPD) values. Analyses were typically performed using the Bayesian Skyline Plot (BSP) model of population growth, which does not use a pre-specified demographic model [43]. We used this model because the VEE complex would not all show the same patterns of demographic change and this did not impose constraints on the different subtypes of VEEV. To estimate substitution rate variation among lineages we used the models implemented in the BEAST program [44] including the uncorrelated lognormal (UCLN) model for the VEEV IE strains and the uncorrelated exponential (UCEX) model for the VEEV ID/II/IAB/IC strains as these were determined to be the most accurate model using the stepping stone algorithm implemented in BEAST [45]. The MCMC chain was 100 million generations long, thinned to include every 5000^th^ generation in the final sample. The program Tracer version 1.5 (http://tree.bio.ed.ac.uk/software/tracer/) was used to confirm convergence and mixing. The software TreeAnnotator version 1.7.1 (http://beast.bio.ed.ac.uk/software/TreeAnnotator) was used to summarize the data output from BEAST. The maximum clade credibility (MCC) tree was estimated using mean node heights after discarding the initial 10% of generations as burn-in.

### Selection analysis

To investigate the nature of selective pressures acting on VEE complex viruses, we estimated the average number of nonsynonymous (*d*_*N*_) and synonymous (*d*_*S*_) nucleotide substitution per site (*d*_*N*_*/d*_*S*_ ratio), using a counting method SLAC [46], as well as the numbers and locations of sites experiencing episodic positive selection using MEME [47].

In the presence of strong purifying selection, standard models of molecular evolution (e.g. GTR +Γ_4_+I) tend to underestimate branch lengths in RNA and DNA virus phylogenies [48–50]. Therefore we re-estimated branch lengths using a Branch-Site REL (BRSEL) [51] model that accounts for variation in selection pressure across the genome and across different branches in the phylogeny, using the HyPhy software package (50). The original formation of the BSREL model allowed three ω (*d*_*N*_/*d*_*S*_) rate classes on each branch, each representing a proportion of sites in the alignment. To prevent over-fitting, we implemented a step-up procedure via an adaptive BSREL model (aBSREL; [52]), starting with a single d_N_/d_S_ class on each branch and testing the fit of an additional d_N_/d_S_ class using small-sample corrected Akaike information criteria (c-AIC).

### Recombination analyses

All sequences were analyzed for recombination using the program RDP3 [53]. Recombination events were determined using RDP, GENECONV, MaxChi, Chimaera and 3Seq, and were only considered robust if they were identified using more than one method.

## Results

### Phylogenetic analysis of the VEE complex strains

**Fig 1:**
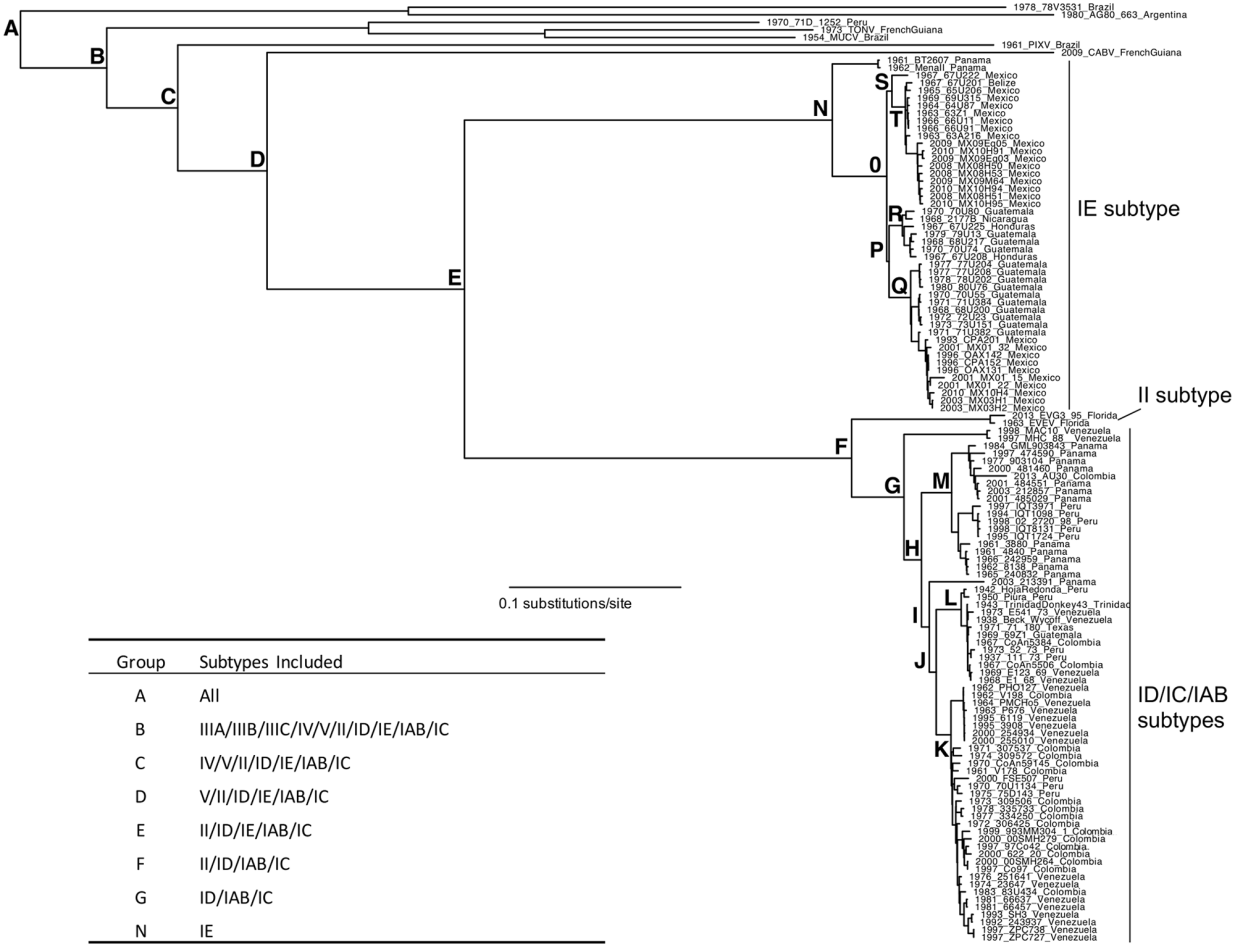
Maximum likelihood phylogeny for VEEV complex. Relevant internal nodes are identified.

The unrestricted Bayesian (MrBayes) and maximum likelihood trees (PAUP*) inferred the expected topology with Cabassou virus (CABV, subtype V), Rio Negro virus (RNV, subtype VI), and Mosso das Pedras virus (MDPV, subtype IF) falling basal to the remainder of the subtypes, as has been shown in previous analyses (Fig 1) [25, 34]. Unexpectedly, the MCC tree, inferred using BEAST (S1 Fig) did not resolve the placement of CABV, RNV, and MDPV, with this grouping showing low posterior support.

Mutations that were unique to each subtype or group of subtypes were identified, as well as those synapomorphies that defined lineages within subtypes; uninformative sites were not counted. For VEEV subtype IE, there were significantly more synapomorphic mutations in the structural protein genes than would be expected if mutations were randomly distributed across the genome (p<0.05, Fisher’s exact test). For all other subtypes and groups of subtypes, the distribution of mutations was not significantly different from expected given the length of the ORF’s. For the VEE complex subtypes III-VI, the number of mutations was identified on each branch (See S1 Fig). There was no correlation between branch length and the number of amino acid substitutions unique to each of these subtypes.

### Correcting for the effect of purifying selection when estimating tMRCAs and evolutionary rates

**Fig 2:**
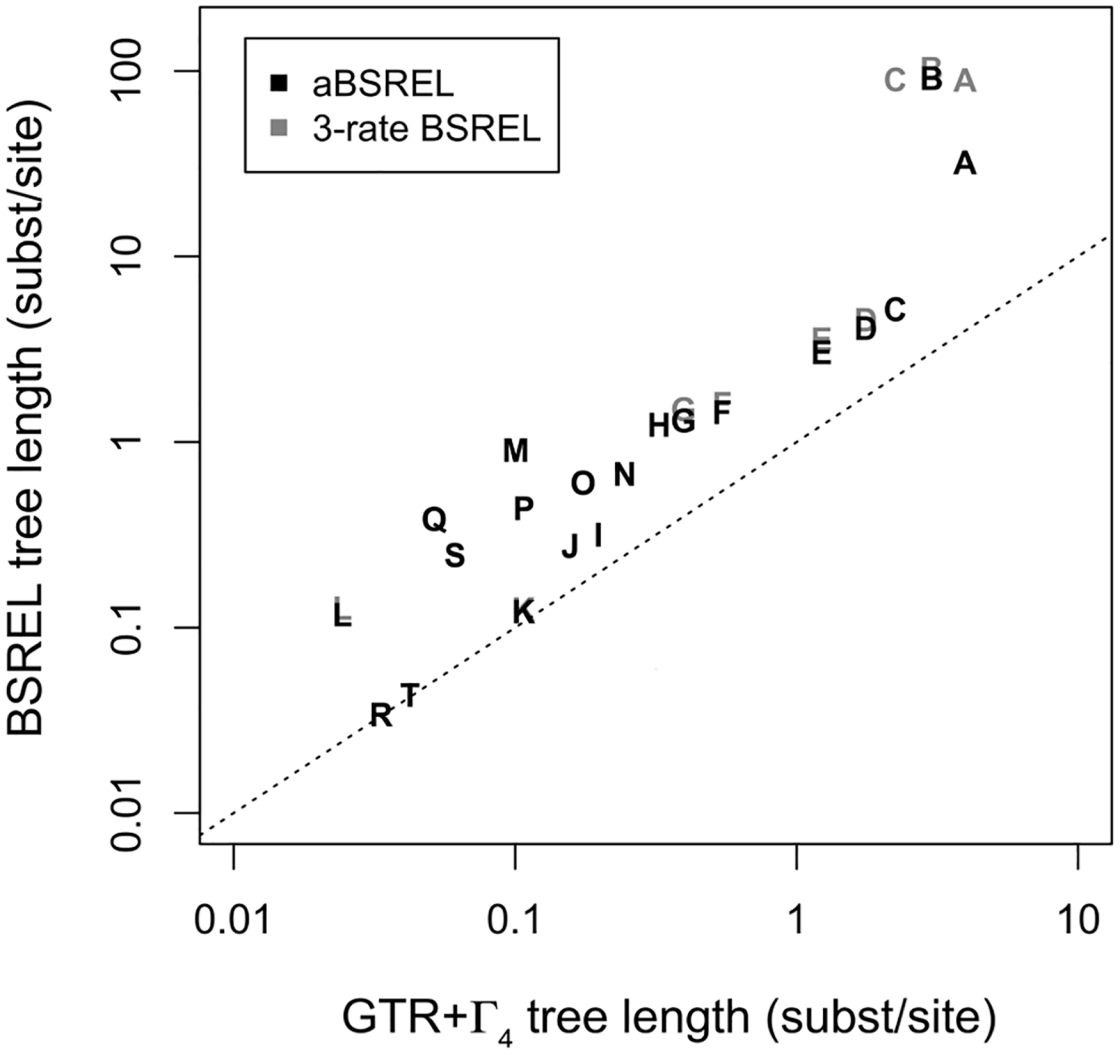
Evidence of branch length underestimation in the VEE complex shown via comparison of tree lengths inferred under Branch-Site REL (BSREL) and GTR+Γ_4_ substitution models. Letters correspond to nodes in maximum likelihood tree. The aBSREL analysis performed with an optimized number of rate classes is shown in black. BSREL analysis performed with a fixed number of three rate classes is shown in gray. Instances in which only a black letter is shown indicate that aBSREL and BSREL produced identical results. The dashed line depicts x = y, an unbiased analysis.

The predominance of purifying selection in alphavirus evolution has been widely reported [54, 55], and this type of selection has been postulated to bias tMRCA estimations for ancient divergence events in RNA viruses [48, 49]. To test our hypothesis that, like other ancient RNA viruses [48–50], dating estimates may be biased in the VEE complex due to the presence of strong purifying selection, we employed a branch-site random effects likelihood (BSREL) approach to determine the extent to which branch lengths (and tMRCAs) were underestimated by standard models of nucleotide evolution. By moving up through the VEE complex phylogeny in an iterative process, we removed the most basal lineages and compared total tree length optimized under BSREL and GTR+Γ_4_, thereby identifying subtrees whose branch lengths did not expand under BSREL compared with a standard nucleotide model (GTR+Γ_4_) (Figs 1 & 2). We assert that it is these subtrees, which were not underestimated by GTR+Γ_4_, whose tMRCA can be reliably inferred by standard Bayesian molecular clock dating (e.g. BEAST) (Fig 2).

**Fig 3:**
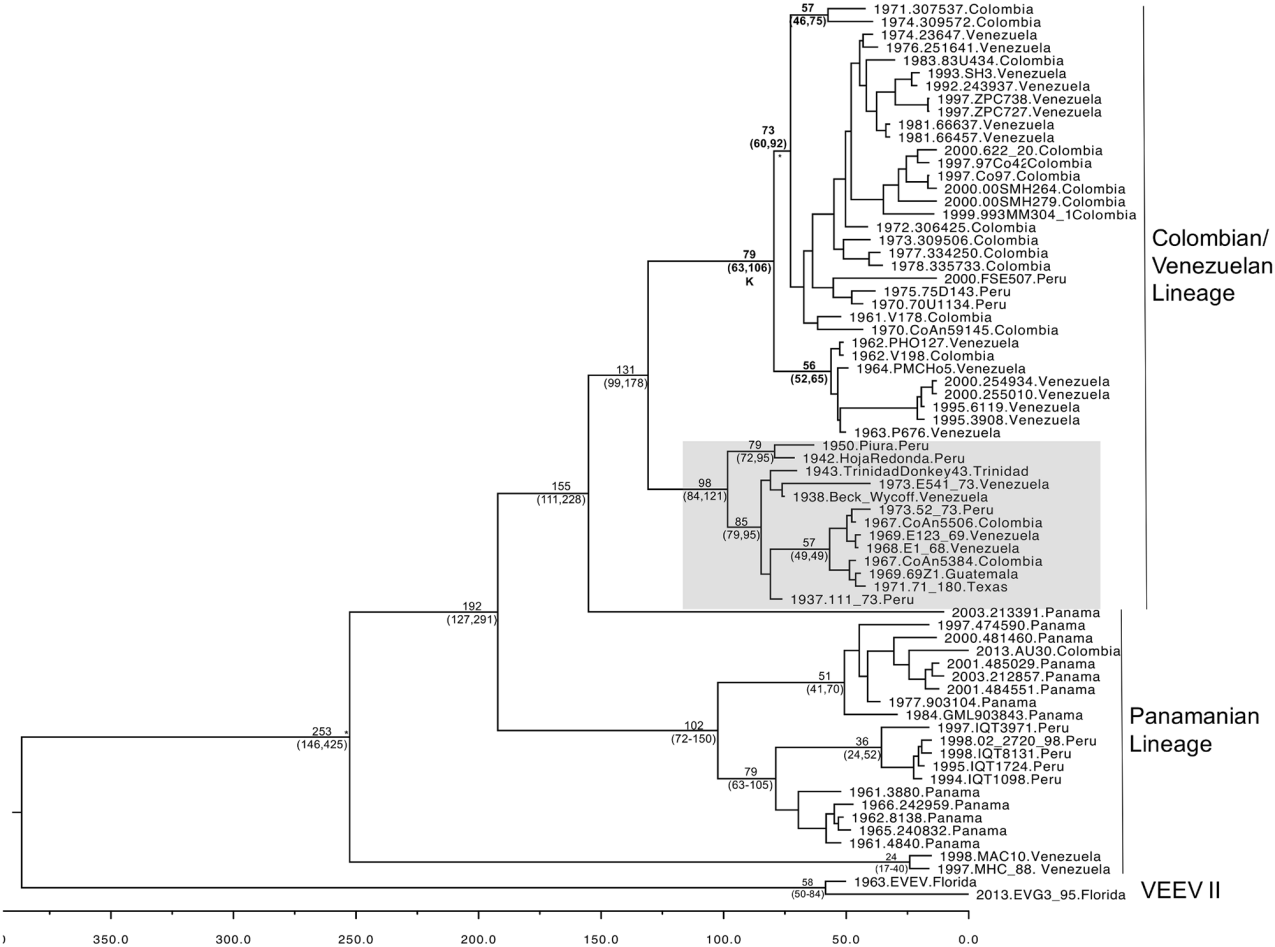
The genetic relationships of the II/ID/IAB/IC subtypes of the VEEV complex. MCC tree as determined by coalescent analysis for the ID/II strains with the IAB and IC strains included. All branches had posterior probabilities of >0.97 except for the branches marked with a *, which did not have significant support. The time to most recent common ancestors (tMRCA) dates for individual branches as identified from the MCC tree are indicated on significant branches with the highest posterior density (HPD) in brackets, dates of clades supported by BSREL were emboldened. Subtype IAB strains are highlighted by the grey box.

As we could not generate a fully resolved MCC tree for the entire VEE complex, two trees were generated for subtypes IAB/IC/ID and for subtypes IE. Removing the other branch lengths removed some of the uncertainty regarding the substitution rates as well as the lack of samples for many of the other subtypes, which can skew the data. The subtype IAB/IC/ID VEEV strains fell into two main groups, a Panamanian lineage and a Venezuelan/Colombian lineage. In addition, we sequenced two additional strains MAC10 and MHC88 from Venezuela, which are outliers from the rest of the ID/IAB/IC strains (Fig 3). These strains showed that VEEV has been circulating in its current form for around 253 years, although the date of this node could not be accurately determined as determined by the BSREL analysis. In fact only group K (Figs 1 and 3), which fell within the Colombian/Venezuelan lineage could be reliably dated at 1934 (1904–1950) (without BSREL correction). The Venezuelan samples MAC10 and MHC88 were basal to the rest of the strains. Within the Venezuelan/Colombian and Panamanian lineages, the Peruvian strains were present in both lineages and, given the shape of the tree and the distribution of these viruses, it is most likely that they represent recent introductions. Although synapomorphic amino acids were identified for the major lineages of the ID subtype (see S2 Fig) the distribution of the amino acid substitutions between the structural and non-structural proteins for each lineage was not significantly different from expected based on a random distribution across the genome.

All of the subtype IAB strains occupied a single clade (see Fig 3), with the Hoja Redonda (1942.HojaRedonda.Peru) and Piura (1950.Piura.Peru) strains forming the most basal branch. Previous analyses suggested that the IAB outbreaks after 1943 were caused by incompletely-inactivated vaccines derived from early IAB isolates [27], consistent with our results. These vaccines were used in South and Central America until the early 1970s, when the TC-83 live-attenuated strain replaced them after its demonstrated efficacy during the 1971 Texas outbreak and in experimental studies [56]. The mechanism of the original IAB emergence is believed to be mutations in the E2 protein gene of enzootic ID strains [19]. Unfortunately, the ID progenitor strains for this group have has not been identified.

**Fig 4:**
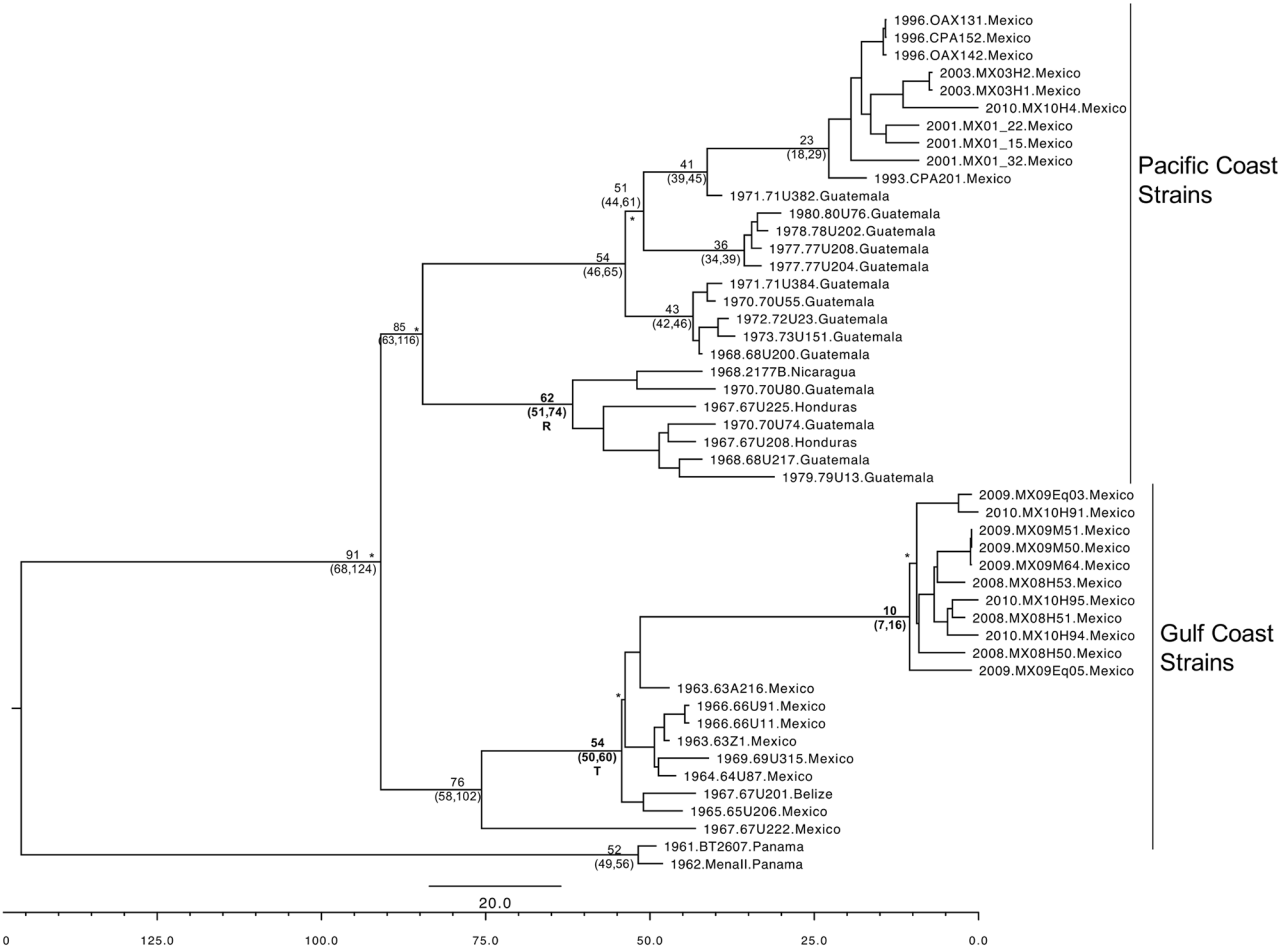
The genetic relationships of the IE subtype of the VEEV complex. MCC tree as determined by coalescent analysis for the IE strains. All branches had posterior probabilities of >0.97 except for the branches marked with a *, which did not have significant support. Time to most recent common ancestor (tMRCA) as determined by the MCC tree were added to major branches with the HPD in brackets. If the clades were supported by BSREL the tMRCA and HPD were emboldened.

For the subtype IE, a separate coalescent analysis was performed (Fig 4). The BT2607 and MenaII isolates were basal in this tree as expected given previous analyses [16]. These viruses were isolated in the 1960s and no further strains are available from this lineage. The remaining subtype IE strains fell into two major groups with the tMRCA of 91 (68–124) years before the present. The Pacific Coast strains contained samples from the entire known geographic range of VEEV subtype IE, except Panama (Guatemala, Honduras, Nicaragua and Mexico) and the Gulf Coast strains included isolates from Mexico only, and a single strain from Belize. The most recent VEEV strains from Mexico were found in this second group. However, the paucity of recent isolates from Central America suggests that some of the temporal groupings in our trees may reflect sampling bias. The Gulf Coast IE strains diverged after the Pacific Coast strains 76 (95% HPD; 58–102) years prior to 2010, and the Pacific Coast strains diverged 85 (95% HPD; 63–116). However, only the clades designated R and T (Figs 1 & 4) could be reliably dated.

### Recombination analysis

There was evidence of VEEV recombination between nucleotides 4800–5830 observed using the program RDP. However, this region corresponds to the nsP3 gene, which is highly variable with frequent insertions and deletions, even within subtypes. Aligning the insertions and deletions is problematic, so this recombination result cannot be confirmed.

### Selection analysis

Using SLAC, we estimated a global d_*N*_/d_*S*_ ratio of 0.057, suggesting that purifying selection is the predominant evolutionary force acting on the VEEV genome. A total of 2900 negatively selected sites were detected using the SLAC algorithm (at p<0.1). Nonetheless, we found evidence for episodic diversifying positive selection at 23 codons using MEME (at p<0.05; identified relative to their position within each protein relative to prototype VEEV strain 3880 in Table 2). Several positively selected codons were detected in important protein genes including the nsP4 RNA-dependent RNA polymerase, Capsid, E2 and E1 glycoproteins. However, these sites did not include substitutions previously demonstrated experimentally to mediate adaptation for equine amplification or bridge vector infection involved in epizootic VEE emergence [21, 57], underscoring the limitations of d_*N*_/d_*S*_-based selection analyses.

**Table 2:** Codon positions predicted to be under episodic positive selection, showing the amino acid changes and the proteins altered. Amino acid position is defined using the Genome sequence 3908 (Accession number U55350 in the NCBI database) and numbered against the non-structural and structural polyproteins.

Protein	Amino acid position (ORF)	p-value	Amino acid change	Protein function
nsP1	3	0.021	K—> T	NA
nsP1	130	0.016	T—> Q/R	Viral methyltransferase
nsP1	444	0.002	N—> P/S/D	NA
nsP1	487	0.049	I—> V/L	NA
nsP2	884	0.007	P—> S	Viral Helicase
nsP3	1344	0.045	Y—> M/I/E/S	Appr -1 -p processing enzyme
nsP4	1921	0.031	S—> V/A	RNA polymerase
nsP4	1991	0.043	L—> E	RNA polymerase
nsP4	2162	0.014	M—> L/A	RNA polymerase
nsP4	2397	<0.001	C—>S/R	RNA polymerase
Capsid	10	0.014	M—> T	Capsid
Capsid	66	0.007	P—> K/Q/R/S	Capsid
Capsid	92	0.025	K—> G/P/Q/R/S	Capsid
**Capsid**^1^	**100**	0.028	**A—>N/T/PV/H/G**	**Capsid**
E3	303	0.006	A—> S/V	E3 glycoprotein
E2	451	0.046	D—> E/G/N/K	E2 glycoprotein
E2	527	0.044	G—> R	E2 glycoprotein
E2	533	0.016	E—> D/K	E2 glycoprotein
E2	547	0.001	T—> K/S/R/Q	E2 glycoprotein
E2	647	0.003	E—> H/N/K	E2 glycoprotein
E1	819	0.038	T—> S	E1 glycoprotein
E1	976	0.020	A—> V	E1 glycoprotein
E1	1153	0.025	K—> R	E1 glycoprotein

^1^Protein change associated with epidemic strains of IE in 1996 and 2003 [58].

## Discussion

VEEV continues to be a major public health problem in Latin America and understanding the evolution and spread of this virus in the New World is integral to implementing surveillance strategies and prevention programs. Although the genetic mechanisms of emergence of epidemic strains is relatively well understood [19, 20, 28, 59], the evolution of the group as a whole has not been studied in a comprehensive manner. Using Bayesian molecular dating techniques, we investigated the evolutionary dynamics of the VEE complex while taking into consideration the effect of purifying selection on the accuracy of dating ancient nodes within this group of viruses. The genomic sequencing of 94 VEEV isolates from all known endemic countries, the inclusion of most isolates from epidemic/epizootic events, and incorporation of the known temporal distribution of available isolates facilitated an extensive investigation into the origin and evolutionary history of this important group of pathogens.

We estimated dates of divergence for several subtypes in the VEE complex. However, the presence of purifying selection can bias such analyses, and our attempts to date the entire VEE complex were limited by this bias. Analysis of ID/IAB/IC strains revealed two main lineages. One circulates primarily in Panama, with a few Peruvian strains included. The origin of this group was estimated at around 102 years ago, although confidence limits were broad. From the phylogeny we were able to infer that circulation of VEEV may have been present initially in Panama, with a subsequent introduction into Peru. The exact mechanism of introduction into Peru is not understood; the enzootic cycle of VEEV involves rodents and mosquitoes that have limited geographic range, suggesting other mechanisms of transport such as birds may be occasionally be involved in virus movement. The presence of Peruvian sequences in two distinct lineages suggests that there have been at least two independent introductions. Exclusion of VEEV subtypes III-VI and the lack of isolates from countries intermediate between Panama and Peru will therefore limit the possibility of fully resolving the ancestral dispersal of these viruses.

As in previous analyses, our results confirm that the IAB strains group together with strain Hoja Redonda, isolated in Peru in 1942, which is basal in the group. Previous partial sequencing and analysis of these strains suggested that some of the IAB outbreaks were a result of incompletely inactivated vaccines [27]. We were able to corroborate this finding using full genome strains that showed tight genetic distance (96.08–99.23%), and no identified ID progenitors (i.e. strains that are genetically related but lack the defining E2 mutations that are associated with the IAB subtypes), although this could be due to inaccurate sampling. However, compared to the spacing of the strains comprising the IC subtype, which are not known to have been used for vaccine production and are interspersed throughout the phylogeny, all the IAB strains appear to have a single origin, and previous analyses have shown that these cluster with vaccine strains used around the time these viruses were circulating [27].

The IC subtype, which has been characterized both phylogenetically and experimentally using reverse genetics, was determined to be a result of amino acid changes in the E2 protein of enzootic subtype ID strains [28, 59]. The IC strains are found within 2 distinct clades in the VEEV phylogeny, particularly in the Colombian/Venezuelan lineage. However, our data suggest that no IC epizootic/epidemic strains have arisen from the Panamanian lineage. It is possible that epistatic interactions limit the emergence of IC epidemic mutations from these Panamanian ID strains, as has been described for chikungunya virus emergence [60, 61]. Despite strong experimental evidence that substitutions in the E2 protein mediate adaptation for equine amplification or bridge vector infection involved in epizootic VEE emergence [21, 57], our sequence analyses failed to detect positive selection on the corresponding codons (Table 2). This finding underscores the limitations of current d_N_/d_S_ methods for identifying unique adaptive substitutions occurring during virus evolution, as they typically require repeated substitution events at a given site to detect adaptive evolution.

The IE VEEV subtype is confined to Central America and Mexico; in fact there appears to be a demarcation between the ID subtype and the IE subtype at the Panamanian/Costa Rican border with the exception of the BT2607 and MenalI strains isolated in western Panama in 1961 and 1962, respectively (Fig 1). More surveillance in Panama is required to determine if these VEEV IE subtypes are still circulating. The main VEEV IE lineage is subdivided into two clades: a widely dispersed group with strains from Guatemala, Honduras, Nicaragua and Mexico (Pacific Coast lineage) and a group with more limited dispersal circulating mainly in Mexico (Gulf Coast lineage). As is expected of a rodent-hosted arbovirus, there is very little spread among countries and the dispersal within the IE subtype appears to occur between neighboring countries, presumably reflecting the limited mobility of rodents and mosquitoes (Fig 4). Given the limited number of countries represented in the IE phylogeny and the lack of recent sampling in most countries, it is hard to draw further conclusions about the historical spread of this subtype within Central America. However, recent detailed studies of VEEV circulation in Mexico demonstrate geographic stability of independently evolving lineages in that country [62].

Previous phylogenetic analyses of the VEE complex viruses were performed using partial and complete structural protein gene sequences [25]. Using these expanded sequences we observed 63 synapomorphic mutations associated with the IE subtype (Group H). Of these, 39 were in the non-structural protein genes and 37 in the structural genes. This finding was significantly different from an expected, random distribution throughout the VEEV genome. It is possible that the preponderance of structural protein substitutions reflects adaptation to different mosquito vectors. Subtype ID strains are vectored by at least three mosquito species: *Cx*. *(Melanoconion) adamesi*, *Cx*. (*Mel*.) *vomerifer* and *Cx*. (*Mel*.) *pedroi* [63], whereas the subtype IE strains appear to be more specialized and vectored nearly exclusively by *Cx*. (*Mel*.) *taeniopus* [64]. Experimental infections with subtypes IAB, IC and ID have demonstrated poor infectivity at the level of midgut infection, suggesting specific subtype IE adaptation to this vector [64, 65]. Although the true distributions of *Cx*. *taeniopus* and *Cx*. *pedroi* are not completely certain because they were not distinguished until 1980 [66], recent collection records and revisions [63, 67–73] suggest that the former is restricted to Mexico, Central America and the Caribbean, while the latter occurs throughout northern South America and Central America, as well as in Mexico. Additionally, neither *Cx*. *vomerifer* or *Cx*. *adamesi*, both subtype ID vectors, are found north of Panama [74, 75], which may restrict the distribution of the ID subtype. Regardless of the original mechanism of spatial compartmentalization, between these subtypes, the IE strains appear to have adapted specifically to *Cx*. *taeniopus* while the other subtypes remain poorly infectious for this species [76].

In summary, our comprehensive analysis of all available full-length VEE complex genomes demonstrated that purifying selection as a confounding factor in coalescent analyses and only the more recently diverged subtypes could have their tMRCAs reliably estimated. Purifying selection is an inherent determinant of arbovirus evolution because of the alternating vector-host transmission and could therefore introduce bias into coalescent analyses of similar viruses. By restricting this bias by analyzing only those clades for which the BSREL analysis indicated that standard nucleotide models would not introduce severe bias we were able to robustly estimate the tMRCA of several VEEV lineages. Evolution of the IE subtype appears to have been characterized by a significant evolutionary shift from the rest of the VEEV complex, with an increase in structural protein substitutions that may reflect adaptation to its mosquito vector. Additionally our inferred tree topologies, suggest that VEEV is maintained primarily in limited geographic foci with only occasional spread to neighboring countries, probably reflecting the limited mobility of rodent hosts and mosquito vectors. Additional strains of subtypes II-VI are needed to more completely characterize the evolution of the entire VEE complex of alphaviruses.

## Supporting information

S1 Table List of virus strains used in the study and known metadata. (PDF)

S1 Fig MCC tree as determined by coalescent analysis for all the VEEV subtypes. Numbers on the branch lengths show the number of unique amino acids that are associated with each particular subtypes.
(PDF)

S2 Fig Unique amino acids associated with each subtype. (PDF)
