# Supplementary material for: Evolution and spread of Venezuelan equine encephalitis complex alphavirus in the Americas
Source: PLoS Negl Trop Dis. 2017 Aug 3;11(8):e0005693. doi: 10.1371/journal.pntd.0005693 (PMC5557581; doi:10.1371/journal.pntd.0005693)
Supplement: S1 Table — (PDF) [file pntd.0005693.s001.pdf]

Supplementary Table 1. Metadata associated with the strains involved in this study. Embolded strains were sequenced for this study

Accession Numbe	Virus Strain	Subtype	Country	District	Collection Date	Host	Passage History
KC344505	69Z1	IAB	Guatemala		1969	Human	sm2, BHK1
KC344483	111/73	IAB	Peru		1973	Horse	sm3
KC344485	CoAn5384	IAB	Colombia	Cali	1967	Horse	sm2,cec1
KC344430	Hoja Redonda	IAB	Hoja Redonda	Peru	1/6/71		CE 1
KC344516	Beck-Wycoff	IAB	Venezuela	Aragua St	1938	Horse	
KR260376*	CoAn5506	IAB	Colombia	Valle del Cauca	20-May-05	Horse	sm1, V1
KU059753*	Piura	IAB	Peru	Piura	1942	Mule	sm3, V1
KU059754*	E1/68	IAB	Venezuela	Guarija	27-Oct-68	Human	Sm1, CEC1, V2
KU059755*	E123/69	IAB	Venezuela	Mara	20-Oct-69	Human	sm1, CEC1, V2
KU059756*	52/73	IAB	Peru	Loreto, La Libertad	1973	Donkey	sm2, V2
KU059757*	E541/73	IAB	Venezuela	Guarija	26-Oct-73	Human	V?
KC344528	PHO127	IC	Venezuela	Zulia St.	1962	Human	v2
KC344484	V178	IC	Colombia	Cuninamarca	1961	Horse	sm1,V1
KC344525	75D143	ID	Peru	Iquitos	1975	Mosquito	cec1 v2
KC344526	IQT1098	ID	Peru	Iquitos	1994	Human	v1
KC344486	70U1134	ID	Peru	Iquitos	1970	Hamster	sm1 cec1
KC344487	334250	ID	Colombia	Puerto Boyaca	1977	Aedes fulvus	v2
KC344488	242959	ID	Panama	Gamboa	1966		
KC344506	4840	ID	Panama		1961	Human	BHK1 Sm2 v1 cec1
KC344523	97CO-42	ID	Colombia		1997		v1
KC344490	IQT1724	ID	Peru	Iquitos	1995	human	v1
KC344502	306425	ID	Colombia	Puerto Boyaca	1972	Hamster	v2
KC344503	903104	ID	Panama	Bayana	1977	Cx. aikenii s.l.	BHK1
KC344504	02-2720-98	ID	Peru	Iquitos	1998	Human	c6/36-1
KC344507	IQT3971	ID	Peru	Iquitos	Unknown	Human	c6/36-1
KC344508	23647	ID	Venezuela	Catatumba, Zulia St.	1974	Hamster	v2
KC344509	251641	ID	Venezuela	Pto Concha	1976	Hamster	v3
KC344510	481460	ID	Panama	Felipillo	2000	Human	v2
KC344511	484551	ID	Panama	Darien, R Iglesias	2001	Human	v2
KC344512	204381	ID	Venezuela	Delta Amacuso	unknown	Unknown	
KC344513	ZPC727	ID	Venezuela	Las Nubes, Catatumba	1997	Hamster	none
KC344514	335733	ID	Colombia	Pto Boyaca	Aug-78	Hamster	none
KC344517	R16905	ID					
KC344518	240832	ID	Panama	Gamboa	1965	unknown	CEC1 v1
KC344519	307537	ID	Colombia	Puerto Boyaca	May-71	Mansonia sp.	vertebrate/mosquito
KC344520	309506	ID	Colombia	Puerto Boyaca	Dec-73	Hamster	vertebrate/mosquito
KC344521	993MM304-1	ID	Colombia	Monte San Miguel	1999	Mosquito	none
KC344522	FSE507	ID	Peru	iquitos	2000	Human	v1
KC344524	CoAn59145	ID	Colombia	Tibu		Hamster	BHK3
KC344471	8138	ID	Panama	El Rincon	1962	Human	CEC-2
KC344472	GML903843	ID	Panama	Bayano	1984	Human	V-1/BHK-1
KC344475	212857	ID	Panama	Darien, Santa Fe	2003	Human	SMB-1
KC344476	213391	ID	Panama	Bocas del Toro,	2003	Human	SMB-1
KC344473	474590	ID	Panama	Mananitas,	1997	Human	V-2
KC344474	485029	ID	Panama	Darien, Yaviza,	2001	Human	V-2
KC344459	00SMH264	ID	Colombia	Monte San Miguel	2000	Mosquito	none
KC344461	00SMH279	ID	Colombia	Monte San Miguel	2000	Mosquito	none
pending*	622-20	ID	Venezuela	Monte San Miguel	2000		
pending*	AU-30	ID	Colombia	Necocli	11-Sep-13	Human	V1
pending*	MAC-10	ID	Venezuela	Padron Agric. Station, Miranda St	20-Jun-05	Hamster	V1
pending*	MHC-88	ID	Venezuela		1998		
KC344527	77U204	IE	Guatemala	La Avellana, Santa Rosa Department	1977		BHK1
KC344468	MX10-H91	IE	Mexico	Minatitlan	August 27,2010	Hamster	original Tissue
KC344492	MX10-H95	IE	Mexico	Minatitlan	August 28,2010	Hamster	original Tissue
KC344515	MX01-32	IE	Mexico	Chiapus State	2001	Hamster	none
KC344431	63Z1	IE	Mexico	Veracruz	8/1963	Human	sm1, V1
KC344480	MX09-M64	IE	Mexico	Tacoteno, Minititlan, Veracruz State	7/2/09	*Cx. taeniopus*	v1
KC344466	71U382	IE	Guatemala	La Avellana, Santa Rosa Department	1971	Hamster	?v1
KC344467	72U23	IE	Guatemala	La Avellana, Santa Rosa Department	1972	Hamster	?v1
KC344437	77U208	IE	Guatemala	La Avellana, Santa Rosa Department	1977	Hamster	
KC344440	66U11	IE	Mexico	Minatitlan, Veracruz State	1966	Hamster	cec1
KC344444	67U225	IE	Honduras	Pto. Cortez	1967	Hamster	sm1
KC344446	65U206	IE	Mexico	Sontecomapan, Veracruz State	1965	Hamster	sm1
KC344448	69U315	IE	Mexico	Sontecomapan, Veracruz State	1969	Hamster	sm1
KC344455	79U13	IE	Guatemala	Izabal Department	1979	Hamster	?
KC344457	63A216	IE	Mexico	Veracruz	8/13-14/1963	Mosquito	sm1
KC344458	70U80	IE	Guatemala	Izabal Department	1970	Hamster	sm1
KC344443	70U74	IE	Guatemala	Puerto Barrios, Izabal Department	8/1970	Hamster	
KC344432	BT-2607	IE	Panama	Almirante	1961	*Cx. taeniopus*	?
KC344433	67U201	IE	Belize		1967	Hamster	sm1
KC344434	73U151	IE	Guatemala	La Avellana, Santa Rosa Department	1973	Hamster	
KC344435	68U200	IE	Guatemala	La Avellana, Santa Rosa Department	1968	Hamster	none
KC344436	70U55	IE	Guatemala	La Avellana, Santa Rosa Department	8/1970	Hamster	sm1
KC344438	78U202	IE	Guatemala	La Avellana, Santa Rosa Department	1978	Hamster	
KC344439	67U222	IE	Mexico	Minatitlan, Veracruz State	1967	Hamster	?,V1
KC344441	2177B	IE	Nicaragua		1968	unknown	V3
KC344442	68U217	IE	Guatemala	Puerto Barrios, Izabal Department	1968	Hamster	?,BHK1
KC344445	64U87	IE	Mexico	Sontecomapan, Veracruz State	7/26-28/1964	*Cx. opisthopus*	sm1
KC344447	66U91	IE	Mexico	Sontecomapan, Veracruz State	4/1966	Hamster	cec1
KC344454	71U384	IE	Guatemala	Santa Rosa Department	1971	Hamster	sm1, V1

**KC344456**	**67U208**	**IE**	**Honduras**		**1967**	**Hamster**	**V?**
**KC344449**	**MX08-H50**	**IE**	**Mexico**	**E. Coachapa, Minatitlan, Veracruz State**	**7/8/08**	**Hamster**	**V1**
**KC344451**	**MX08-H53**	**IE**	**Mexico**	**Tacoteno, Minatitlan, Veracruz State**	**7/13/08**	**Hamster**	**V1**
**KC344464**	**MX03-H2**	**IE**	**Mexico**	**Las Coaches, Pijijiapan, Chiapas State**	**3-Aug-03**	**Hamster**	**V1**
**KC344482**	**MX03-H1**	**IE**	**Mexico**	**Chiapus State**	**2003**	**Hamster**	**V1**
**KC344465**	**MX10-H4**	**IE**	**Mexico**	**El Dorado, Mapastepec, Chiapas State**	**25-Jul-10**	**Hamster**	**V1**
**KC344450**	**MX08-H51**	**IE**	**Mexico**	**E. Coachapa, Minatitlan, Veracruz State**	**7/9/08**	**Hamster**	**V1**
**KC344492**	**MX10-H94**	**IE**	**Mexico**	**Minatitlan, Veracruz State**	**Aug-10**	**Hamster**	**V1**
**KC344478**	**MX09-M51**	**IE**	**Mexico**	**Tacoteno, Minititlan, Veracruz State**	**9/20/09**	***Cx. nigripalus***	**V1**
**KC344479**	**MX09-M50**	**IE**	**Mexico**	**Tacoteno, Minititlan, Veracruz State**	**9/20/09**	***M. titillans***	**V1**
**KC344481**	**MX09-Eq03**	**IE**	**Mexico**	**Tacoteno, Minititlan, Veracruz State**	**10/27/09**	**Horse**	**V1**
**KC344463**	**MX09-Eq05**	**IE**	**Mexico**	**Tacoteno, Minititlan, Veracruz State**	**28-Oct-09**	**Horse**	**V1**
**pending***	**MX01-15**	**IE**	**Mexico**		**2001**	**Hamster**	
**KR260737***	**EVG3-95**	**II**	**United States**	**Everglades National Park, Florida**	**2-Jul-13**	**Mosquito**	
AF069903	71_180	IAB	United States	Texas	1971	horse	
L01442	Trinidad Donkey	IAB	Trinidad	N/A	1943	N/A	N/A
NC_001449	P676	IC	Venezuela	Miranda State	1963	horse	
AF004459	243937	IC	Venezuela	Trujillo State	9/1/93	human	V1
AY973944	254934	IC	Venezuela	Barinas	10/4/00	horse	
AY986475	255010	IC	Venezuela	Barinas	02/20/2000	horse	
U55342	V198	IC	Colombia	La Guajira Dept.	1962	human	
U55345	PMCH05	IC	Venezuela	Monagas State	1964	human	
U55347	6119	IC	Venezuela	Falcon State	05/24/1995	human	
U55350	3908	IC	Venezuela	Sinamaica, Zulia State	09/16/1995	human	
U55360	SH3	IC	Venezuela		9/1/93	human	
AF004458	66637	ID	Venezuela	Sinamaica, Zulia State	11/19/1981	hamster	
AF004458	66457	ID	Venezuela	Sinamaica, Zulia State	11/11/81	hamster	
AF100566	ZPC738	ID	Veneuzela	Zulia State	9/24/1997	hamster	
AY966912	IQT8131	ID	Peru		1998	human	
L00930	3880	ID	Panama	Canito	1961	human	
U55362	83U434	ID	Colombia	N. Santander	Jun-1983	hamster	SM1, V1
AF075252	MenaII	IE	Panama		1962		
AF448535	CPA152	IE	Mexico	Chipas	1996	horse	
AF448536	OAX131	IE	Mexico	Oaxaca	1996	horse	
AF448537	CPA201	IE	Mexico	Chiapas	1993	horse	
AF448538	OAX142	IE	Mexico	Oaxaca	1996	horse	
AF448539	80U76	IE	Guatemala	La Avellana, Santa Rosa Dept.	1980	Hamster	
AY823299	MX01-22	IE	Mexico		2001	N/A	
AF075257	78V3531	IF	Brazil		1978	N/A	
AF075251	EVEV	II	United States	Florida	1963		
AF075254	MUCV	IIIA	Brazil		1954	N/A	
AF075253	TONV	IIIB	French Guiana		1973	Mosquito	
AF075255	71D-1252	IIIC	Peru		1971	Hamster	
AF075256	PIXV	IV	Brazil		1961		
AF075259	CABV	V	French Guiana		1968		
AF075258	AG80-663	VI	Argentina		1980		
EF151502	FL93-939	I	USA	Florida	1993		
DQ241303	PE_3.0815	II	Peru		1996	Mosquito	
DQ241304	PE_0.9155	III	Peru		1996	Mosquito	
EF151503	BeAr436087	IV	Brazil				
