# Supplementary figures and images for: Evolution and spread of Venezuelan equine encephalitis complex alphavirus in the Americas
Source: PLoS Negl Trop Dis. 2017 Aug 3;11(8):e0005693. doi: 10.1371/journal.pntd.0005693 (PMC5557581; doi:10.1371/journal.pntd.0005693)

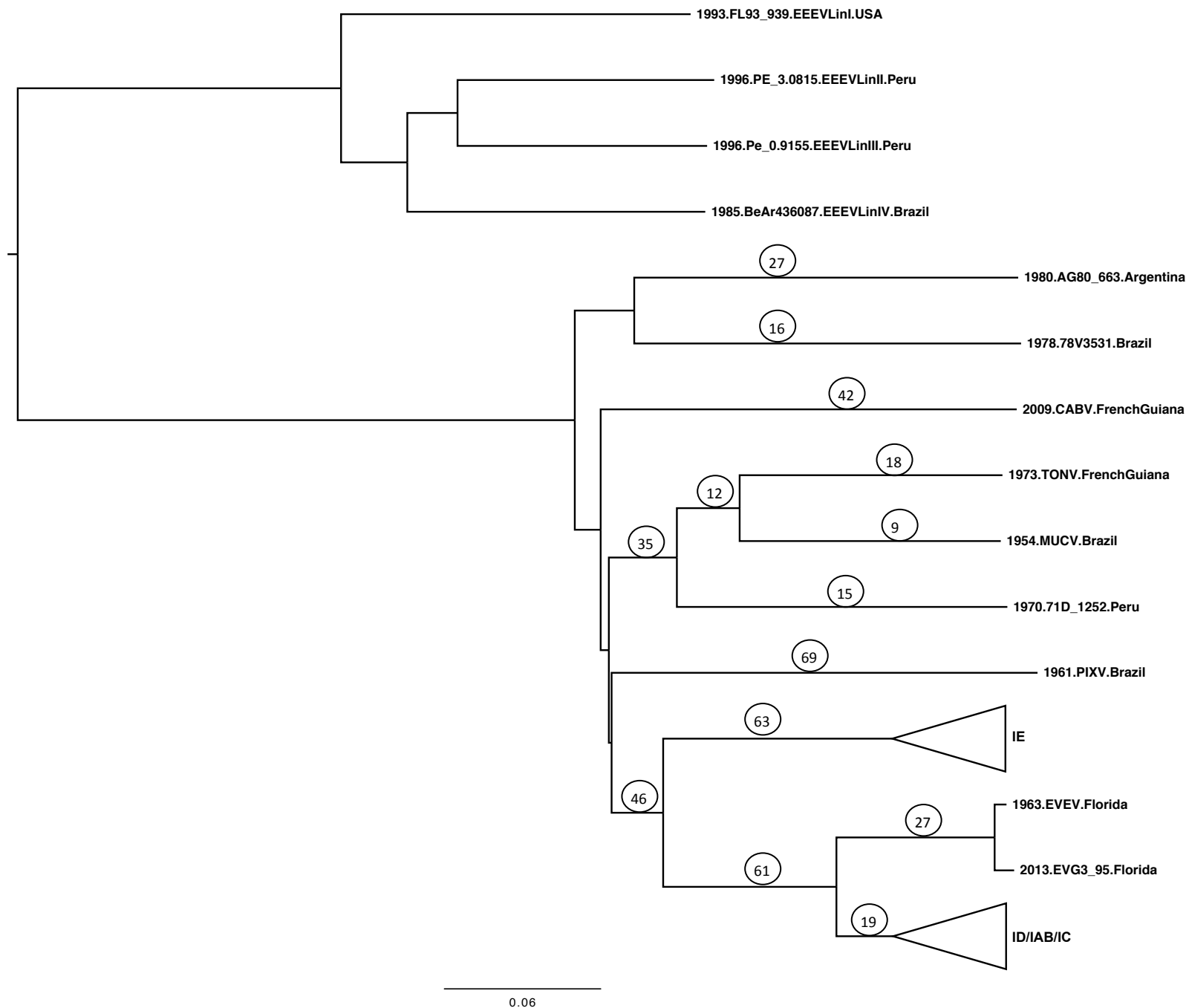

Supplement: S1 Fig — Numbers on the branch lengths show the number of unique amino acids that are associated with each particular subtypes. (PDF) [file pntd.0005693.s002.pdf]
