# Supplementary material for: Evolution and spread of Venezuelan equine encephalitis complex alphavirus in the Americas
Source: PLoS Negl Trop Dis. 2017 Aug 3;11(8):e0005693. doi: 10.1371/journal.pntd.0005693 (PMC5557581; doi:10.1371/journal.pntd.0005693)
Supplement: S2 Fig — (PDF) [file pntd.0005693.s003.pdf]

Supplemental Figure 2. List of all amino acid changes associated with major subtypes.

AG80

Position	Amino Acid
98	Ser
111	Asn
124	Thr
128	Ileu
232	Phe
237	ser
459	Ala
491	Thr
495	Val
1000	Gln
1192	Ser
1219	Gln
1431	Gln
1877	Leu
1878	Ala
1932	Arg
2376	Ser
2591	Ileu
2697	Ala
2706	Lys
2795	Thr
2935	Thr
3232	Lys
3245	Leu
3412	Val
3514	Ileu
3793	Val

78V

Position	Amino Acid
98	Ser
102	Arg
111	Asn
132	Ser
176	Val
238	Ser
305	Ser
1876	Gly
1955	Ser
2376	Val
2471	His
2696	thr
3163	Met
3307	Leu
3435	MET
3796	Ileu

AG80/78V

Position	Amino Acid
200	Ser
203	Val
228	Met
239	Ileu
428	Val
434	Lys
458	Thr
472	Met
498	Arg
569	Lys
584	Pro
779	Tyr
786	Val
1651	Asp
1767	Ileu
1859	Ser
2290	Ala
2590	Val
2720	Gln
3129	Val
3174	Gly
3351	Cys
3564	THr
117	Glu

CABV

Position	Amino Acid
76	Asn
111	Gly
114	Gln
116	Leu
124	Gln
125	Glu
127	Ser
144	Asp
147	Ileu
239	Ileu
269	Thr
299	Thr
403	Asp
440	Val
514	Thr
610	Thr
661	Gly
738	Lys
750	Ileu
779	Tyr
873	Gln
976	Glu
1000	Asn
1179	Thr
1701	Ser
1771	Asp
1813	Ala
1913	Ala
1942	Ser
1950	Ileu
2048	Asp
2290	Cys
2577	Ser
2590	Ileu
2609	Thr
2706	Ileu
2709	Ala
3259	Asn
3355	Leu
3424	Ileu
3435	Phe
3793	Thr

71D

Position	Amino Acid
227	Val
409	Glu
465	Lys
466	Ser
750	Leu
1871	Ileu
1942	THR
1950	Cys
1990	Arg
2141	Thr
3039	Val
3146	Ala
3218	Lys
3477	Ser
3713	Ileu

MUCV

Position	Amino Acid
81	Val
85	Arg
94	Tyr
239	Ileu
661	Thr
1942	Pro
1989	Arg
2048	Asn
2709	Val

TONV

Position	Amino Acid
75	Asn
98	Ser
495	Thr
513	Met
661	Asn
663	His
705	Arg
750	Thr
761	Asp
1118	Phe
1771	Gln
1913	Ser
2048	His
2618	Leu
2709	Asp
3326	Ileu
3477	Val
3514	Ala

TONV/MUCV

Position	Amino Acid
162	Ileu
353	Glu
508	Ileu
643	Ileu
978	Thr
1867	Leu
1990	Lys
2577	His
2935	Ala
3044	Val
3383	Phe
3793	Leu

TONV/MUCV/71D

Position	Amino Acid
124	Glu
440	Val
514	Val
582	Glu
758	Lys
764	Ileu
804	Ileu
976	Glu
1179	Thr
1195	Lys
1384	Ser
1524	Phe
1810	Val
1813	Gln
1814	Gly
1861	Asp
1862	Ileu
1866	Ala
2163	Met
2290	Ser
2591	Thr
2618	Val
2707	Ala
3131	Asp
3174	Ser
3175	Ala
3346	Val
3486	Arg
3518	Pro
3564	ser
3627	Val
3653	Leu
3752	Ser
3796	Val
3804	Leu

TONV/MUCV/71D

Position	Amino Acid
3161	Thr
3163	Lys
3164	Thr
3174	Ala
3200	Met
3268	Gly
3324	Met
3351	Ala
3355	Ala
3521	Arg
3594	Val
3595	Ala
3646	Arg
3729	Thr

PIXV

Position	Amino Acid
98	Ser
104	Ser
106	Ala
117	Thr
144	Ser
146	His
147	Ileu
167	Glu
203	Ser
236	Thr
237	Asp
239	Ileu
253	Glu
271	Ser
386	Ileu
408	Met
471	Met
491	Arg
501	Thr
505	Ala
508	Glu
644	Asn
661	Glu
673	Arg
683	His
761	Asn
786	Val
830	Tyr
873	Pro
974	Ala
1000	Ala
1022	Arg
1206	Ileu
1794	Asp
1810	Ser

PIXV

Position	Amino Acid
1813	Ser
1815	Val
1850	Ileu
1857	Val
1859	Gly
1860	Pro
1862	Val
1867	Ileu
1871	Gln
1877	Ileu
1879	Val
1921	Leu
1922	Pro
1980	Leu
2191	Thr
2290	Thr
2376	Leu
2616	Asp
2697	Gln
2699	Arg
3161	Thr
3163	Lys
3164	Thr
3174	Ala
3200	Met
3268	Gly
3324	Met
3351	Ala
3355	Ala
3521	Arg
3594	Val
3595	Ala
3646	Arg
3729	Thr

VEEV ID

Position	Amino Acid
133	Met
424	Arg
1683	Gly
1698	THR
1704	Ileu
1739	Leu
1772	Ileu
1781	Ser
1781	Gln
1867	Val
1876	Thr
1922	Thr
1934	Leu
2056	Gln
2704	Lys
2706	Gly
2729	Ala
2945	Val
3655	Ser

EVEV

Position	Amino Acid
133	Val
256	Lys
337	Ileu
417	Leu
466	Asn
500	Ala
656	Arg
750	Thr
887	Gly
974	Ser
986	Thr
1735	Gly
1739	Ser
1763	Arg
1861	Thr
1922	Ileu
1936	Leu
2694	Ser
2709	Arg
2715	Glu
3154	Leu
3260	Tyr
3338	Thr
3412	Thr
3531	Ala
3655	Leu
3793	Ser

VEEV IE

Position	Amino Acid
94	Phe
121	Glu
140	Thr
143	Phe
232	Phe
424	Arg
475	Pro
514	Ser
574	Thr
610	Gln
656	Arg
664	Glu
871	Asp
872	Ser
954	Lys
976	Asp
1150	Ala
1179	Ser
1191	Asp
1219	Gln
1299	Phe
1547	Met
1660	Ala
1668	Ala
1670	Asn
1778	Ala
1794	Arg
1815	Ser
1857	Asp
1859	Ileu
1860	Ser
1935	Leu
2257	Lys
2290	Asp
2301	Ileu
2437	Arg
2458	Met
2608	Glu
2619	Ser
2704	Gly
2714	Lys
2715	Leu
2716	Val
2731	Gly
2903	Ser
2966	Gly
3053	Val
3163	Gln
3200	Thr
3229	Val
3263	Phe
3274	Ala
3309	Ser
3355	Val
3363	Ileu
3424	Ala

VEEV IE

Position	Amino Acid
3481	Val
3531	Ser
3564	Val
3625	Lys
3690	Tyr
3869	Ileu

VEEV ID/II

Position	Amino Acid
85	Arg
98	Thr
425	Arg
431	Ileu
475	Lys
492	Cys
569	Lys
683	Thr
870	Lys
886	Thr
1040	Glu
1147	Leu
1150	Asn
1154	Gln
1206	Val
1285	Ser
1431	Lys
1444	Ser
1451	THr
1479	Met
1484	Ala
1641	Gln
1651	Ala
1686	Glu
1696	Gln
1778	Pro
1812	Ileu
1866	Ser
1889	ser
1955	Val
1957	Thr
2091	Ala
2290	Asn
2380	Arg
2412	Ala
2471	Gly
2577	Leu
2608	Lys
2670	Thr
2698	Ala
2701	Lys
2702	Gln
2708	Gln
2713	Lys
2714	Asn
2720	Ala
2726	Asn
2959	Ala
2960	Val
3119	Ala
3121	Gln
3192	Lys
3218	Ala
3231	Ala
3233	Gly
3271	Asp

VEEV ID/II

Position	Amino Acid
3274	His
3349	Val
3564	Leu
3593	Val
3698	Ala

VEEV ID/IE/II

Position	Amino Acid
225	Met
240	Leu
270	Gln
441	Val
444	Asn
455	Ileu
461	Glu
474	Glu
551	Ala
554	Asp
615	Gln
643	Thr
663	His
693	Asp
738	Lys
758	Lys
760	Lys
804	Ileu
805	Arg
940	Thr
978	Thr
993	Arg
1118	Tyr
1174	Glu
1527	Ser
1564	Val
1657	Lys
1737	Ileu
1779	Ser
1810	Leu
1862	Leu
2096	Gln
2356	Ileu
2634	Pro
3042	Thr
3101	Asn
3377	Met
3441	Val
3477	Pro
3491	Val
3504	Met
3520	Thr
3661	Ser
3678	Ala
3713	Thr
3804	Ileu
